# Measles immunity in medical center staff after changes in national and local hospital vaccination policies

**DOI:** 10.1186/s12879-022-07419-x

**Published:** 2022-05-04

**Authors:** Meng-Yu Lin, Hsin-Hui Shao, Meng-Ting Tsou

**Affiliations:** 1 Lienchiang County Hospital, Lienchiang County, Taiwan ROC; 2 The Department of Family Medicine, Mackay Memorial Hospital, Taipei City, Taiwan ROC; 3 Nursing, and Management, Mackay Junior College of Medicine, Taipei City, Taiwan ROC

**Keywords:** Measles, Immunization, Hospital personnel, Seroprevalence

## Abstract

**Background:**

Measles vaccination was introduced in Taiwan in 1978, and the disease was declared eliminated in Taiwan in 2007. However, new cases have been reported unpredictably since then. Hospital medical staff are at particularly high risk for measles. We evaluated the immunity status of hospital medical staff after changes in national and local hospital vaccination policies.

**Methods:**

This retrospective study was conducted in a tertiary care medical center from January 2008 to June 2018. Data were retrieved from all healthcare workers receiving employment medical examinations. Those with a full medical record including the geometrical mean titer (GMT) of anti-measles IgG were included. Age and sex differences in the GMT were analyzed by Student’s t-tests and Chi-squared tests. Univariate and multivariate logistic regression analysis were used to determine the odds of immunity.

**Results:**

The IgG positive rate increased with age group (p < 0.001). Seropositive rates for the birth before 1977 and after 1978 groups were 94.8% and 70.2% (p < 0.001). The odds ratio was also significantly different between both cohorts (1.000 vs. 0.423, p = 0.002). Staff in the examination department showed the lowest positive percentage of 70.3% (95% CI: 66.9–73.7%), whereas staff in preventive and long-term care services disclosed the highest positive percentage of 83.2% (95% CI: 76.1–90.2%). Subgroups 2015, 2017, and 2018 (p = 0.046, 0.046, 0.049), after the vaccination booster policy was launched, showed significant increases in seropositivity.

**Conclusions:**

Immunity efficacy is better in birth groups before 1977, which was highly related to natural infection before national policy launched. The policy of vaccination is an effective method, but medical staff attains inadequate protective antibody levels for maintenance of herd immunity. A pre-employment policy of screening a third booster vaccine of measles (or MMR) is recommended to lower the incidence of disease spreading and avoid outbreaks.

## Background

Measles is a highly contagious respiratory disease. Nine out of ten susceptible individuals with close contact with an infected patient will develop measles [1]. The disease is spread through air, droplets, or by contact with nasopharyngeal mucus from infected individuals, and can result in severe complications, including death [1]. Measles was once common in Taiwan. More than 99% of children were affected, and epidemic outbreaks occurred roughly every 2 years. Fortunately, it has been brought under control in Taiwan after a nationwide routine vaccination policy was adopted in 1978. The annual incidence of measles in Taiwanese was reduced to less than 1/1,000,000 during 2003–2008. However, new outbreak clusters still emerge sporadically [2]. It is important to monitor the immunity status of the hospital staff in order to reduce the risk of nosocomial infection [2–4].

Our hospital started routine pre-employment screening of all staff in 2008, with measles, mumps, and rubella (MMR) vaccine boosters required for anyone with undetectable antibody titers since 2012. This study evaluated the effects of the implementation of national and hospital vaccination policies on measles seroprevalence among healthcare workers (HCWs) in Taiwan. It was hoped that these data would help guide the development of a local screening program.

## Methods

### Study population

The study was performed at Mackay Memorial Hospital, a 2000-bed tertiary care hospital in Northern Taiwan, a region with an estimated population of 2.67 million. The study data were obtained from routine pre-employment physical examinations of HCWs between January 2008 and June 2018, which included assessment of measles antibodies. Since 2008, routine testing for measles antibodies was performed for all staff in this medical center and MMR boosters were required for those with undetectable antibody titers from 2012. Participants included were full-time HCWs at least 18 years old with at least one measles IgG titer result in their medical records. There were no other exclusion criteria nor sampling selection in this study. All participants including doctors, nurses, examination department, preventive and long-term care services, and administration were divided into six age groups (18–20, 21–30, 31–40, 41–50, 51–60, and 61–70 years of age). We evaluated the effect of hospital policy enforcement by comparing seropositivity before 2012 with subsequent years.

### Laboratory values

A serum sample was collected from each HCW for assessment of anti-measles-virus immunoglobulin G by a quantitative measles IgG enzyme-linked immunosorbent assay (LIAISON^®^ XL, Japan). Sensitivity and specificity were 98.42% (95% CI = 96.25–99.31%) and 93.94% (95% CI = 79.83–99.34%), respectively. Seropositivity was defined as titer ≥ 165 mIU/mL, while titer < 135 mIU/mL was considered negative. Titers between 135 and 165 were considered equivocal and the test was repeated. If titers still range between 135 and 165, the data were considered negative at the end.

### Statistical analysis

All participants’ age, sex, and anti-measles IgG titer information was collected in a FileMaker Pro (www.filemaker.com) database. SPSS 24.0 (IBM Corp., Armonk, NY, USA) was used for analysis. Geometrical mean titers (GMTs) were calculated by age and sex groups for further comparison. Age and sex differences in the GMT were analyzed by Student’s t-test, and Chi-squared tests were applied to compare differences in the proportions of subjects positive for anti-measles antibody. Confidence intervals of seroprevalence were estimated by large sample method. Correlation between age and GMT was determined by linear regression analysis. Univariate and multivariate logistic regression analysis were used to determine the odds of immunity associated with age, sex, vocation, and being subject to government and hospital vaccination policies. P-values of < 0.05 were considered significant and confidence interval (CI) of 95% was assumed.

### Ethics statement

This serosurvey was reviewed and approved by the Ethics Committee of Mackay Memorial Hospital (No. 18MMHIS103). Informed consent was waived by the same ethics committee that approved the study (Institutional Review Board of Mackay Memorial Hospital).

## Results

**Fig. 1:**
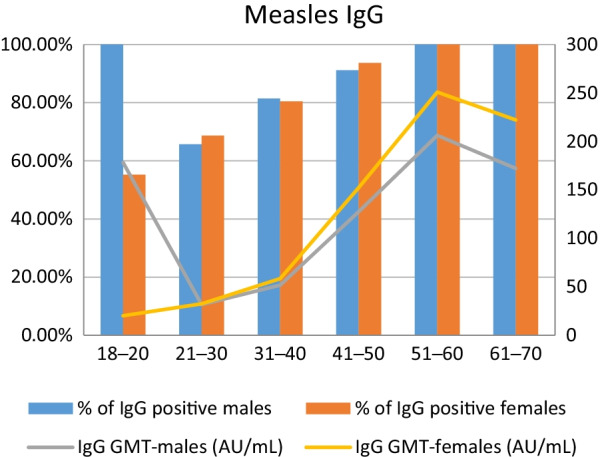
Proportion of Measles IgG positive subjects and Measles IgG GMT, by age group and gender. Orange columns: % of Measles IgG positive females. Blue columns: % of Measles IgG positive males. Yellow line: Measles IgG GMT in females (AU/mL). Gray line: Measles IgG GMT in males (AU/mL)

A total of 2905 participants were evaluated during annual pre-employment health screenings dating from 2008 to 2018 in a tertiary hospital center. 2111 participants were female with the mean age of 28.78 ± 8.92 (SD) years, and the mean age of men was 29.09 ± 7.13(SD) years. The percentages (numbers) of subjects aged 18–20, 21–30, 31–40, 41–50, 51–60, and 61–70 years were 1.38% (40), 68.02% (1976), 19.86% (577), 6.99% (203), 2.89% (84), and 0.86% (25), respectively. (Table 1) Of 2905 participants, 2128 (73%) had positive IgG, and the rate showed a positive correlation with increasing age group (Table 2). Similar correlations were found in both male and female subgroup analysis (Fig. 1).

**Table 1:** Age and sex groups of hospital employees

Age group	Female		Male		Total	
	n	%	n	%	n	%
18–20	38	1.80	2	0.25	40	1.38
21–30	1428	67.65	548	69.02	1976	68.02
31–40	394	18.66	183	23.05	577	19.86
41–50	158	7.48	45	5.67	203	6.99
51–60	76	3.60	8	1.01	84	2.89
61–70	17	0.81	8	1.01	25	0.86
Total	2111		794		2905	

Chi-square = 32.199; p < 0.001

**Table 2:** Seropositivity in different age groups

Age group	n	Positive	%	95% CI		P-value
18–20	40	23	57.50	42.18	72.82	< 0.001
21–30	1976	1341	67.86	65.80	69.92	
31–40	577	466	80.76	77.54	83.98	
41–50	203	189	93.10	89.61	96.59	
51–60	84	84	100	100.00	100.00	
61–70	25	25	100	100.00	100.00	
Total	2905	2128	73.25			

Staff in the examination department (median age: 28.11years) showed the lowest percentage of positive rates, at 70.3% (95% CI: 66.9–73.7%), where staff in preventive and long-term care services (median age: 34.91years) had the highest percentage of positive rate: 83.2% (95% CI: 76.1–90.2%) (P = 0.004). (Table 3) Odds ratio of different subgroups including sex, age, the impact of policy, and vocation were calculated. By regression analysis, there was no significant difference in gender and vocation. People born after 1977 have 0.423 times the odds to be seropositive relative to people born before 1977. Subgroup analysis comparing the 18–20 age group with other age groups revealed significant differences (p = 0.001) except for the 21–30 age group (p = 0.174) (Table 4).

The odds ratio comparing the data before 2012 with subsequent years showed significant differences in subgroup 2015, 2017, and 2018 (p = 0.046, 0.046, 0.049) with a pattern of improvement after the hospital booster vaccination policy was launched (Table 5).

**Table 3:** Crude seroprevalence by vocation (N = 2905)

Vocation	Total (N)	Positive (n)	Median (IQR)	Seroprevalence in % (binominal 95% CI)	P-value
Nurses	1016	722	22.52 (4.11)	71.1 (64.3–77.9)	0.004
Doctors	735	537	26.66 (6.92)	73.1 (69.3–76.9)	
Examination	242	170	28.11 (9.00)	70.3 (66.9–73.7)	
Preventive and long term care service	131	109	34.91 (20.82)	83.2 (76.1–90.2)	
Administration	781	596	28.53 (10.60)	76.3 (72.9–79.7)	

**Table 4:** Multiple logistic regression on final immune status (N = 2905)

Exposure variable	Odds ratio	P-value	95% CI	
			Lower	Upper
Sex				
Female	1.000	–	–	–
Male	0.864	0.178	0.699	1.069
Impact of national policy				
Up to 12/31/1977	1.000	–	–	–
Beginning 01/01/1978	0.423	0.002	0.214	0.695
Age-group				
18–20	1.000			
21–30	1.567	0.174	0.821	2.978
31–40	3.041	0.001	1.538	6.014
41–50	9.846	0.001	2.627	36.910
51–60	12.155	0.001	2.261	42.963
61–70				
Vocation				
Nurses	1.000	–	–	–
Doctors	1.177	0.204	0.916	1.512
Examination	0.975	0.044	0.315	0.998
Preventive and long-term care service	1.261	0.368	0.761	2.090
Administration	1.092	0.464	0.862	1.384

**Table 5:** Regression analysis of immune status after hospital booster vaccine policy

Exposure variable	Odds ratio	P-value	95% CI	
			Lower	Upper
Year				
2003–2012 (before hospital policy)	1.000	–	–	–
2013	1.129	0.779	0.484	2.632
2014	1.013	0.715	0.423	2.486
2015	2.036	0.046	1.283	4.216
2016	1.098	0.815	0.502	2.401
2017	1.680	0.046	1.001	2.667
2018	2.088	0.049	1.100	4.360

## Discussion

According to the recommendation from Advisory Committee on Immunization Practices and the United States Centers for Disease Control and Prevention, two doses of MMR vaccine are considered an effective method for preventing measles infection [1, 2, 5]. The incidence of measles was reduced in Taiwan after the introduction of MMR vaccination by the government in 1978. There were fewer than 100 cases in 1993 and fewer than 10 in 2007 [5, 6]. Elimination was then declared.

However, sporadic measles outbreaks still have been reported, not only in communities but also in hospitals [4–6]. Most cases were imported, but occasionally hospital cluster outbreaks were seen. Hospitals may become an infection focus when this occurs, resulting in serious consequences. Hospital staff is a particularly high-risk group. Expansion of vaccination policies for health care workers has been emphasized in other studies [5–11].

In our study, the Measles IgG positivity rate and titers showed a mostly positive correlation with age group. Lower positive seroprevalence is found in the under-40 groups. These results are compatible with previous measles serological studies in Taiwan during 1995–1997 and 2004–2009. Immunity apparently waned over time after widespread vaccination was introduced in 1978, this could be due to the difference in exposure between infection-acquired and vaccine-acquired before and after the introduction of the vaccination program. This finding is consistent with previous reports from Taiwan and other countries [3, 6, 11, 12]. In age subgroup regression analysis, a significant immunological difference was seen between the 18–20 and over-30 groups. Comparison between the immunity of HCWs born before 1977 and after 1978 appeared significant, too. This suggests natural infection provides a longer immunity duration than acquired immunity from vaccination [3].

Through the subgroup analysis via vocation, we found that preventive and long-term care service had a median age of 34.91 years with interquartile range (IQR) of 20.82, had the greatest seroprevalence of 83.2%; whereas the examination staff had a median age of 28.11 years with IQR of 9.00, had a positive rate of only 70.3%. Although the seropositivity differences in vocations may be confounded by age, the tendency of low seropositivity of examination staff makes continuous monitoring and revaccination of second-line HCWs a reasonable measure.

According to the World Health Organization (WHO), immunity prevalence of 93–95% is enough to achieve herd immunity [13]. The seropositivity rate of HCWs from a medical center located in southern and northern Taiwan was 81.1% and 85.8%, respectively [3, 14]. The seropositivity in our HCWs was 73%, although vaccine coverage had been 95% for more than a decade. Compared with other countries’ reported rates of immunity, 91–93% in France, 86% in Italy, and 73% in Korea, our results were at the lower end of the range, and demonstrated insufficient immunity to interrupt measles transmission [11, 15–17]. It is important for us to assess our HCWs’ measles-antibody status and give boosters to those without enough protection. HCWs under 40 especially need to be evaluated. This has been found to be one of the most cost-effective ways to prevent nosocomial outbreaks [5, 18].

Our hospital started routine pre-employment screening for measles antibody in 2008. Only measles-specific antibody detectable from the serological test was considered positive. Staff who had undetectable measles antibody received booster vaccinations since 2012. Differing from previous studies in Taiwan or other countries, we followed up measles positive rate on HCWs every year after the launch of hospital policy [3, 11, 17]. The results after regression analysis had a trend of positive change and were statistically significant in 2015, 2017, and 2018. The reason for a lack of significance in other years could be related to the different health examination follow-up intervals of the hospital, thus not all HCWs were included every year. This evidence could serve as guidance for hospital policies in the future.

There are limitations to our study. First, this is a single-center, retrospective study. It may not represent the situation in other hospitals or nationwide. Second, we could not confirm whether our HCWs had ever received boosters before. Third, we did not recheck measles antibody after the vaccine booster. Finally, there were only two subjects in the age 18–20 male group, meaning our findings may not be applicable to this age group.

## Conclusions

Public health effort is an effective way to prevent measles outbreaks. However, our results indicated that medical staff maintain inadequate protective antibody levels to maintain herd immunity. Evidence of immunity was deemed valid when staff had received two doses of measles vaccines, had laboratory evidence of immunity or disease, or had diagnosis of measles infection from a healthcare provider.

A pre-employment policy of screening, followed by a third booster vaccine for measles (or MMR) for seronegative individuals, is recommended. The policy would reduce the likelihood of outbreaks among HCWs. Immunity rates were higher in birth groups before 1977, consistent with prior research finding that natural exposure to infection yields more prolonged protection than acquired immunity from vaccination. Screening not only new staff but all HCWs may be a more effective way in reducing the possibility of nosocomial infection among HCWs, patients, and hospital visitors.

## Data Availability

The datasets used and/or analyzed during the current study are available from the corresponding author on reasonable request.

## Abbreviations

GMT: Geometrical mean titer
MMR: Measles, mumps and rubella
HCWs: Healthcare workers
SD: Standard deviation
IQR: interquartile range
